# Comparative Serological Study for the Prevalence of Anti-MERS Coronavirus Antibodies in High- and Low-Risk Groups in Qatar

**DOI:** 10.1155/2019/1386740

**Published:** 2019-02-18

**Authors:** Reham A. Al Kahlout, Gheyath K. Nasrallah, Elmoubasher A. Farag, Lingshu Wang, Erik Lattwein, Marcel A. Müller, Mohamed E. El Zowalaty, Hamad E. Al Romaihi, Barney S. Graham, Asmaa A. Al Thani, Hadi M. Yassine

**Affiliations:** ^1^Department of Biomedical Sciences, College of Health Sciences, Qatar University, Doha, Qatar; ^2^Biomedical Research Center, Qatar University, Doha, Qatar; ^3^Communicable Diseases Control Programs, Public Health Department, Ministry of Public Health, Doha, Qatar; ^4^Vaccine Research Center, National Institute of Allergy and Infectious Diseases, National Institute of Health, Bethesda, MD, USA; ^5^Euroimmun AG, Luebeck, Germany; ^6^Institute of Virology, Charité - Universitätsmedizin Berlin, Charitéplatz 1, Berlin, Germany; ^7^Virology and Microbiology Research Laboratory, School of Health Sciences, College of Health Sciences, University of KwaZulu-Natal, Westville Campus, Durban 4000, South Africa

## Abstract

Infection with Middle East respiratory syndrome coronavirus (MERS-CoV) could be asymptomatic or cause mild influenza-like illness. Therefore, the prevalence of MERS-CoV infections in the general population could be underestimated, which necessitates active surveillance to determine the epidemiological importance of asymptomatic cases. The aim of this study is to evaluate the performance of various serological assays and to estimate the seroprevalence of anti-MERS-CoV antibodies in high- and low-risk groups in Qatar. A total of 4858 samples were screened, including 4719 samples collected from healthy blood donors (BD) over a period of five years (2012-2016), 135 samples from baseline case contacts (CC) collected from individuals in close contact with three positive PCR-confirmed patients (CP), and four samples from MERS-CoV CP. Initial screening using anti-MERS-CoV IgG (IgG rS1-ELISA kit) revealed ten reactive samples from BD (10/4719, 0.21%), one from CC (1/135, 0.74%), and three from CP (3/4, 75%). Samples from CP but not from BD were also reactive by whole-virus anti-MERS-CoV IgG (*n* = 3/4) and IgM (*n* = 1/4) indirect immunefluorescent tests (IIFT) and pseudoparticle neutralization test (ppNT). The reactive sample from CC was also confirmed by ppNT. Surprisingly, one out of thirteen (7.7%) randomly selected IgG rS1-ELISA-negative BD samples from the initial screening was reactive by the IgM-IIFT (but not by the IgG-IIFT) and was subsequently confirmed by ppNT. All IgG rS1-ELISA-reactive samples from BD exhibited considerable reactivity to the four circulating human coronaviruses (HKU1, OC43, 229E, and NL63). Cross-reactivity with SARS was only reported for samples from CP using IgG and IgM-IIFT. In conclusion, we report a low prevalence of anti-MERS antibodies in the general population, which coincides with the low number of all reported cases by the time of our study (2017) in Qatar (*n* = 21). The false-positive results and the observed cross-reactivity between MERS-CoV and other circulating human coronavirus necessitate more detailed evaluation of available serological assays.

## 1. Background

Middle East respiratory syndrome coronavirus (MERS-CoV) is a human beta-coronavirus (HCoV) that is originally identified in the Kingdom of Saudi Arabia (KSA) in 2012. So far, the WHO has reported 2229 cases of MERS-CoV infections in 27 countries, with a fatality rate of about 36% (*n* = 791) [1].

MERS-CoV-specific antibodies are widely found in dromedary camels (*Camelus dromedarius*) along with viral shedding of similar viruses detected in human. Accordingly, dromedaries are considered the primary source of MERS-CoV transmission to humans, although the original source for the virus is still unknown [2–4].

Phylogenetic analysis groups coronaviruses into four genera: alpha-, beta-, gamma-, and delta-coronaviruses. Bats are considered the natural reservoirs of these viruses. Although SARS-CoV is closely related to bat CoV (BtCoV) HKU3, and MERS-CoV is closely related to Pipistrellus BtCoV HKU5 and Tylonycteris BtCoV HKU4, the serologic and antigenic relationship between these viruses is unclear. Generally, coronaviruses across subgroups demonstrate a low level of cross-reactivity for the S protein and limited preservation of cross-neutralizing epitopes [5, 6]. However, few studies have demonstrated cross-reactivity among these Betacoronavirus. It has been shown that mouse hyperimmune sera to SARS-CoV harbor low levels of neutralizing activity against MERS-CoV [5]. Further, sera samples from SARS patients demonstrated 60.7% (17/28) binding and 25% (7/28) neutralizing activities to MERS-CoV, suggesting cross-reactivity within subgroup viruses [7].

Following its first isolation, several laboratory diagnostic tests for MERS-CoV have been developed [8–11]. Molecular tests such as RT-PCR and sequencing are majorly used in diagnosing MERS-CoV infections [12]. The United States Centers for Disease Control and Prevention (CDC) limits the use of serological tests for investigational or surveillance settings and not for diagnosis [13]. They established a two-phase serological test approach to detect anti-MERS antibodies based on ELISA (targeting S1 antigen) followed by whole-virus IgG and IgM IIFT and microneutralization test for confirmation. The microneutralization assay is highly specific and it is the gold standard for measuring specific neutralizing antibodies against MERS-CoV in sera samples. Nonetheless, compared with the ELISA and IIFT, the microneutralization assay requires a BSL3 facility, which is not available at many places, and it is labor-intensive and time-consuming, requiring at least 5 days before results are available [13, 14].

In the State of Qatar, twenty-one cases have been reported until 2017, including seven deaths (33.3%). Interestingly, 95% (*n* = 20) of the cases in Qatar were reported in males compared to only one female case. Thirteen of the MERS cases were reported in camel farm owners and workers, and five were suspected human-to-human transmissions, of which three were nosocomial infections (Ministry of Public Health-Qatar, personal communication).

Qatar was the first nation to report on the isolation and full genome sequencing of MERS-CoV from camels [3]. In a separate study from Qatar, Reusken et al. reported that ~ 7% (20/294) of persons with camel contact have antibodies reactive with MERS-CoV S1 antigen, compared to zero reactive in control or noncase contact samples. Using 90% plaque-reduction neutralization test (PRNT90), only 10 of the 20 (5%) MERS-CoV S1 antibody-reactive samples were confirmed positive [15].

Due to the uncertain epidemiological picture of MERS-CoV among Qatar population, we designed a staged serologic surveillance study for MERS-CoV consisting of initial screening by anti-MERS-CoV IgG rS1-ELISA kit followed by evaluation of reactive samples using whole-virus indirect immunofluorescence assays (IgM- and IgG-IIFT) and ppNT. We also tested the cross-reactivity of IgG rS1-ELISA-reactive samples with the four circulating human coronaviruses using ELISA and IIFT. This study targeted three groups: (i) low-risk group constituted of 4719 samples obtained from blood donors (BD) collected over a period of five years (2012-2016), (ii) high-risk group represented by 135 samples obtained from baseline case contacts (CC) collected from individuals who were in close contact with confirmed cases during the acute phase (first week), and (iii) four samples from PCR-confirmed MERS-CoV patients (CP). The high-risk group is defined by the individuals that were in direct contact with the confirmed cases either at work, house, or hospital (medical staff), prior or after symptom development. Our findings suggest that MERS-CoV is not heavily circulated among the population of Qatar. Additionally, the presence of antibody responses to other human coronaviruses resulted in false-positive results in binding assays, which mandate the need for more evaluation studies of the currently available diagnostic serological assays.

## 2. Methodology

### 2.1. Patient Samples

In total, 4858 plasma samples were analyzed in this study. Samples were distributed as follows: 4719 plasma samples were collected from BD during previous studies [16–20] over a period of five years (2012-2016; age: 19-88 years; mean age 37 years), 135 plasma samples were collected from individuals that were in CC to four CP (age: 14-49 years; mean age 31 years), and four plasma samples were collected from CP (age: 30-70 years; mean age 52). The CC individuals represented the patient's family members, healthcare workers, and camel farm coworkers. Samples from CC were collected within the first week of the patient's admission to hospital. This study was approved by Qatar University-IRB Review Exemption No. QU-QU-IRB 622-E/16.

### 2.2. Serological Testing

Initially, all plasma samples were screened for the presence of anti-MERS-CoV (S1 subunit) IgG using a commercial IgG rS1-ELISA kit (rS1-ELISA, Euroimmun, cat no. EI 2604-9601G). Since samples from CC were collected within the first week of primary case identification, these samples (*n* = 135) were also tested for the presence of IgM antibodies using whole-virus anti-MERS-CoV IgM IIFT kit (IgM-IIFT) (Euroimmun, cat no. FI 2604-1010 M). The anti-MERS-CoV (IgM/IgG) IIFT is based on MERS-CoV-infected eukaryotic cells and the anti-MERS-CoV ELISA (IgG) on purified S1 antigens of MERS-CoV. As recommended by the WHO, all borderline and reactive samples were then tested for the presence of anti-MERS-CoV antibodies using whole-virus indirect immunofluorescence assay (IgM- and IgG-IIFT) (Euroimmun, cat no. FI 2604-1010). Further, the borderline and reactive samples in addition to 13 randomly selected IgG rS1-ELISA-negative samples (served as negative controls) were screened with an in-house recombinant-S1 protein IIFA IgG (rS1-IIFA; Institute of Virology, Charité - Universitätsmedizin Berlin, Germany; as described by Corman et al. 2012 [8]) in order to reduce the possibility of cross-reactivity of human sera with the full MERS virus antigens presented by Vero cells in whole-virus IIFT. Final confirmation was performed using pseudoparticle neutralization test (ppNT) against two MERS-CoV strains, the EMC strain (GenBank JX869059) and the Jordan N3 strain (GenBank KC776174), as previously described [21]. The determination of cross-reactivity of borderline and reactive samples against other human coronaviruses was performed using: (i) commercially available whole-virus IgM/IgG IIFT for SARS-CoV (Euroimmun, cat no. FI 2601-1010 G/M), (ii) prototype whole-virus IgG IIFT kit for HCoV-229E (Euroimmun, prototype kit), (iii) in-house ELISA for HKU1-CoV using recombinant S1 protein (Sino Biological Inc., catalog # 40021-V08H), and (iv) IgG rS1-IIFT for all other human-CoV (Institute of Virology, Charité - Universitätsmedizin Berlin, Germany) [22].

## 3. Results

**Figure 1:**
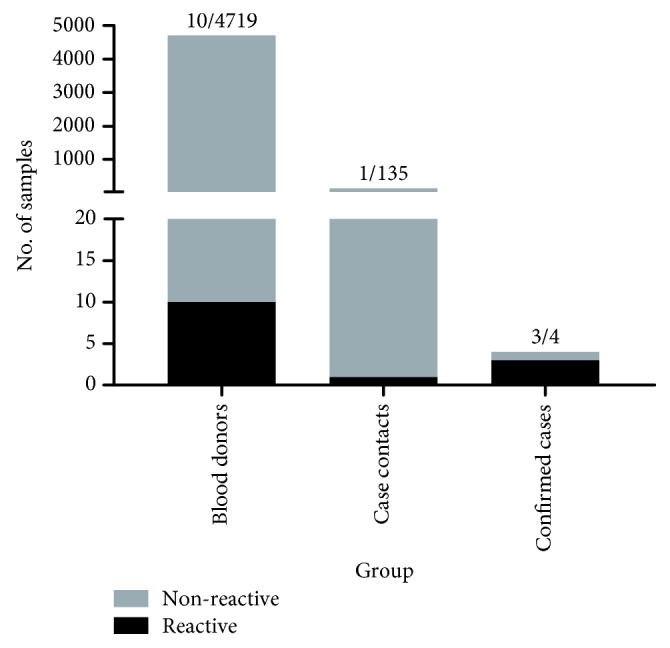
Number of reactive samples using rS1-ELISA (IgG) screening. A total of 4858 plasma samples were initially screened for anti-MERS S1 IgG using rS1-ELISA. The graph shows the number of reactive samples in three groups: blood donor (*n* = 4719), case contacts (*n* = 135), and confirmed cases (*n* = 4).

The demography and characteristic profiles of the study population are summarized in Table 1. Initial screening for anti-MERS-CoV antibodies using IgG rS1-ELISA revealed 10/4719 (0.21%) and 1/135 (0.74%) reactive samples from BD (three borderline and seven positive samples) and CC, respectively. On the other hand, 3/4 CP (75.0%) were reactive with IgG rS1-ELISA assay (Figure 1; Table 2). Since CC samples were collected within the first week of primary case identification, samples were also tested by IgM-IIFT and all were negative.

As recommended by the WHO, borderline and reactive samples were then tested for the presence of anti-MERS-CoV IgG using whole-virus and recombinant (r) S1-IIFT. Analysis with whole-virus IgG-IIFT confirmed only two (2/10) samples from BD as well as three (3/4) samples from CP. Interestingly, none (0/10) of the above BD reactive samples tested positive with neither rS1-IIFT nor ppNT assay. The positive IgG rS1-ELISA CC sample was only tested by ppNT and it was positive. All of the randomly selected IgG rS1-ELISA negative BD samples were also negative by whole-virus and rS1 IgG-IIFT (Tables 3 and 4).

To determine the status of infection (recent versus older), all IgG rS1-ELISA reactive samples were further evaluated for the presence of IgM antibodies as an indication for recent infections using whole-virus IIFT (IgM-IIFT), and only one was marginally reactive and that was from a CP (Table 4). Strikingly, one of the 13 randomly selected IgG rS1-ELISA IgG-negative BD samples from the initial screening was found reactive for IgM antibodies (using IgM-IIFT) with a titer of 320. Positivity of this sample was further confirmed with ppNT, with EC50 titer of 500 (Table 3). The sample was obtained from a 35-year-old Syrian citizen residing in Qatar.

Discrepancies in the results obtained from different binding assays could be due to cross-reactivity with other viruses. Hence, we evaluated the cross-reactivity of rS1-ELISA-reactive samples for IgG antibodies against all currently known human coronaviruses. All tested BD samples including the negative controls from the initial screening exhibited reactivity to at least 3 of 4 human coronaviruses. All rS1-ELISA-reactive samples were reactive to the four seasonal coronaviruses: 229E, HKU1, OC43-CoV, and NL63 (Table 5). The reactivity was also high in the negative controls from the initial screening reaching 100% (13/13) for 229E, 92% (12/13) for HKU1 and OC43, and 84% (11/13) for NL63 (partial data is shown in Table 5). None of the tested BD samples were reactive to SARS-CoV using whole-virus or rS1-IIFT IgG. Similarly, all samples from CP were also highly reactive with other human coronaviruses. Interestingly, two of the CP samples had considerable reactivity to SARS-CoV with titers of 320 and 3200 using IgG rS1-IIFT (Table 5).

Discrepancies in cross-reactivity were also observed among different serological tests for human coronaviruses. For example, one sample from CP tested negative with whole-virus IIFT IgG for HCoV-229E, but it was reactive with recombinant S1 protein of the same virus using similar assay. Similarly, two samples from CP showed reactivity to SARS-CoV in the IgG rS1-IIFT, whereas only the sample with higher antibody titer reacted with the whole-virus IgG-IIFT assay. Further, all samples from CP reacted with HKU1 spike protein in rS1-ELISA, but only two samples yielded positive reaction with the IgG rS1-IIFT (Table 5).

## 4. Discussion

Qatar reported a relatively low number of MERS cases in comparison to neighboring countries despite the fact that MERS-CoV continues to circulate in camels [23, 24]. In the absence of a clear epidemiological view of MERS-CoV, we present here a comparative serological study for the prevalence of anti-MERS coronavirus antibodies in high- and low-risk groups in Qatar.

Following the WHO recommendation, we run initial screening for IgG antibodies using rS1-ELISA, and reactive samples were then confirmed with full virus and rS1-IIFT IgG, followed by ppNT. Combined results from different serological tests indicate the low presence of neutralizing anti-MERS-CoV antibodies in the general population in Qatar (1/4719), while the rate increases to 1 : 135 in the high-risk group (CC). Our results revealed a few interesting observations. First, the only confirmed positive sample from the BD group was accidentally detected when we tested 13 randomly selected negative samples (originally selected to serve as a negative control) from the initial screening (using rS1-ELISA-IgG) for IgM response, where screening for IgM is not typically done in similar studies [14]. This mandates the development and utilization of assays that measure both classes of the antibodies for screening processes. Nonetheless, our results coincide with the low number of reported cases in Qatar (*n* = 21) compared to the neighboring countries such Saudi Arabia that has the highest number of reported MERS cases worldwide (*n* > 1700) [25, 26]. In a similar study using a similar approach in Saudi Arabia, 15/10009 (0.15%) were confirmed positive for anti-MERS antibodies in the general population. These numbers are slightly higher than what we observed in our study (1/4719; 0.02%); however, the low number of positive samples in both studies prevents a significant statistical analysis [14].

Another interesting observation was the seropositivity in CC samples. Although those samples were collected within the first week of primary case identification, only one sample was positive for IgG but not for IgM antibodies, indicating previous exposure to the virus. It also confirms that infection with MERS-CoV can go unnoticed and that surveillance studies shall be done systematically to include screening for IgM and IgG responses. Similar to the above observation in the general population, the rate of seropositivity in the high-risk group (CC) in our analysis was lower (1/135; 0.74%) than that observed in Saudi Arabia (7/227; 3.08%). Several factors could explain the difference between both studies including the difference in sample size as well as the demographics and livestock population in both countries.

One out of four MERS CP samples did not show antibody response using rS1-ELISA, whole virus, and rS1-IIFT assays. The sample was collected during the acute infection phase, which explains the absence of anti-MERS-CoV IgG response. A recent study from South Korea indicated that humoral response to MERS-CoV, as measured by binding and neutralizing assays, wean rapidly after one year of infection and becomes undetectable in about 67% of those who show mild illness upon infection [27]. In another study from Korea, it was shown that none of the asymptomatically infected individuals showed seroconversion; however, the seroconversion rates gradually increased with increasing disease severity reaching 60.0%, 93.8%, and 100% in symptomatic infection without pneumonia, pneumonia without respiratory failure, and pneumonia progressing to respiratory failure, respectively [28]. Such studies indicate that human humoral immune response to MERS-CoV is a complicated phenomenon that requires further investigation. It further implies that the infection rate with MERS-CoV in the Middle East could be underestimated and that the fatality rate associated with MERS-CoV infection is most likely overestimated.

As observed in other studies, the rS1-ELISA resulted in significant false-positive results. While 10/4719 (0.2%) samples were positive in this assay, only 2/10 (20%) were positive with whole-virus IgG-IIFT and null were confirmed with rS1-IIFT and ppNT (0%). Similarly, out of 10009 tested samples in the Saudi Arabia study, 152 were positive with rS1-ELISA (1.5%), 17 of which (11%) were positive with whole-virus IIFT and 15 were confirmed with neutralization assay [14]. These results indicate cross-reactivity between MERS-CoV and other human coronaviruses. To test this further, we employed several binding assays to test the reactivity of our samples with five known human coronaviruses, namely, SARS, HKU1, 229E, OC43, and NL63 CoV. Interestingly, all MERS-reactive samples from first screening, as well as most of the randomly selected negative control samples, had cross-reactivity to at least 3/4 seasonal human viruses, but none of them were cross-reactive with SARS-CoV. On the other hand, two of the CP samples were cross-reactive with SARS-CoV with intermediate titers. Accordingly, it is not clear which of the human coronavirus is inducing this cross-reactivity with MERS-CoV, which mandates further investigation. We also observed discrepancies in cross-reactivity when using different assays, where rS1-based IIFT were more sensitive than full virus counterparts. That could be due to the higher concentration of the specific S1 protein in the first assay, which also ensures the use of defined regents (purified proteins) for developing screening assays.

On the other hand, cross-reactivity between SARS and MERS-CoV has been reported earlier. A 2013 study by Chan et al. indicated that 17/28 (60.7%) of SARS patients had significant binding antibody titers (using IIFT), of which seven (25%) had anti-MERS (EMC) neutralizing antibodies at low titers, which significantly correlated with that of HCoV-OC43 [7]. In the same study, bioinformatics analysis demonstrated a significant B-cell epitope overlapping the heptad repeat-2 region of spike protein between the two viruses [7].

We acknowledge few limitations to our study including the use of ELISA procedure to screen for anti-MERS-CoV IgG response, dominance of males over females (4642 versus 77), and non-Qataris over Qataris (3791 versus 928), as well as the screening at one-time point. Our findings affirm on the use of neutralization assay, and to a lower extent spike protein-specific IIFT, as confirmatory tests considering the high cross-reactivity among different human coronaviruses. Such findings were similar to what has been recently reported by Drosten et al. who showed that excess in IgG detection by anti-MERS rS1-ELISA IgG was not confirmed with IgG-IIFT assay [29].

## 5. Conclusion

Our results indicate a high discrepancy between different assays available in the market to screen for anti-MERS-CoV antibodies. ELISA-based assay seems to be more prone to produce false-positive results, while results of IIFT should always be confirmed by neutralization test. Most of the available ELISA assays utilize S1 protein, which is part of the whole protein and in many cases is not well characterized before use. Using affinity purified and structurally defined protein might be more reliable to produce accurate results. The recent determination of several ectodomain structures from many coronaviruses should be instrumental to design better immunogens [30–36], which can also be utilized to develop more specific screening assays. Lastly, further investigation of the possibility of MERS-CoV transmission through a blood transfusion from asymptomatic donors should also be assessed, though no cases have been reported so far with evidence of blood transfusion as a source of MERS-CoV infection.

## Figures and Tables

**Table 1:** Characteristic profile of the study population.

	BD (2012-2016)			CC (2015-2016)			CP (2015-2016)		
	Number	Age range (mean)	Exposure	Number	Age range (mean)	Exposure (*n*)	Number	Age range (mean)	Exposure
Qatari males	906	19-88 (37)	Unknown	11	14-49 (31)	Family contact (37), healthcare worker (73), camel farm worker (25)	2	29-69 (51)	Camel farm
Qatari females	22			3			0		
Non-Qatari males	3736			93			2		
Non-Qatari females	55			28			0		
Total	**4719**			**135**			**4**		**4858**

**Table 2:** Number of reactive samples for anti-MERS S1 IgG using rS1-ELISA.

Sample source	Year of collection (no. screened)	No. borderline/no. screened (%)	No. reactive/no. screened (%)
BD	2012 (120)	*1/120 (0.83)*	0/120 (0)
	2013 (28)	0/28 (0)	0/28 (0)
	2014 (611)	0/611 (0)	*1/611 (0.16)*
	2015 (3383)	*1/3383 (0.03)*	*5/3383 (0.15)*
	2016 (577)	*1/577 (0.17)*	*1/577 (0.17)*
Subtotal	4719	*3/4719 (0.08)*	*7/4719 (0.13)*
CC	May-2015 (100)	0/100 (0)	*1/100 (1)*
	Feb-2016 (10)	0/10 (0)	0/10 (0)
	June-2016 (25)	0/25 (0)	0/25 (0)
Subtotal	135	0/135 (0)	1/135 (0.74)
CP	Mar-2015 (1)	0/1 (0)	*1/1 (100)*
	May-2015 (1)	0/1 (0)	*1/1 (100)*
	Feb-2016 (1)	0/1 (0)	*1/1 (100)*
	May-2016 (1)	0/1 (0)	0/1 (0)
Subtotal	4	0/4 (0)	*3/4* (75)
Total	**Total (4858)**	**3/4858 (0.06)**	**12/4858 (0.25)**

Positive samples are shown in *italic*.

**Table 3:** Comparative serological analysis of reactive and borderline samples from blood donors (BD).

		rS1-ELISA^∗^	Full virus IIFT		rS1-IIFT	ppNT
	Sample identifier	IgG (OD, ratio, endpoint titer)	IgG titer	IgM titer	IgG titer	IC50 titer (EMC/JordanN3)
Reactive (*n* = 7)	BD 2014/597	*(0.905, 2.114, 201)*	0	0	0	<50
	BD 2015/1303	*(0.397, 1.3, 101)*	*10000*	0	0	<50
	BD 2015/3004	*(0.439, 1.26, 201)*	0	0	0	<50
	BD 2015/3119	*(0.402, 1.06, 401)*	0	0	0	<50
	BD 2015/3380	*(0.477, 1.1, 101)*	*10000*	0	0	<50
	BD 2015/3513	*(0.497, 1.39, 401)*	0	0	0	<50
	BD 2015/4435	*(0.661, 1.74, 201)*	0	0	0	<50
Borderline (*n* = 3)	BD 2012/2644	*(0.333, 0.83, 101)*	0	0	0	<50
	BD 2015/1816	*(0.456, 0.823,201)*	0	0	0	<50
	BD 2015/4708	*(0.408, 1.07, 101)*	0	0	0	<50
Selected negative (showing 3/13) ^∗∗^	BD 2015/2859	(0.034, 0.076, <101)	0	0	0	<50
	BD 2015/2988	(0.039, 0.112, <101)	0	0	0	<50
	BD 2015/3379	(0.065, 0. 16, <101)	0	*320*	0	*531/502*

^∗^Initial screening was done with rS1-ELISA, and reactive samples were further tested with various serological assays as indicated above. ^∗∗^13 negative samples from the initial screening with rS1-ELISA were selected for comparison, and one was found positive with full virus IgM and ppNT. Positive samples are shown in *italic*.

**Table 4:** Comparative serological analysis of reactive samples from CC and CP.

	rS1-ELISA	Full virus IIFT		rS1-IIFT	ppNT
Sample identifier	IgG (OD, ratio, endpoint titer)	IgG titer	IgM titer	IgG titer	IC50 titer (EMC/JordanN3)
CC May.2015	*(0.61, 1.5, 101)*	Quantity not sufficient	Quantity not sufficient	Quantity not sufficient	*76/149*
CP Mar.2015	*(1.084, 2.86, 201)*	*10000*	0	*>10000*	*630/1707*
CP May.2015	*(0.412, 1.37, ND^∗^)*	*3200*	0	*320*	*199/688*
CP Feb.2016	*(2.229, 6.517, ≥ 3201)*	*>32000*	*100*	*>10000*	ND

Positive samples are shown in *italic*. ND: not determined. ^∗^This samples showed controversial results in rS1-ELISA IgG and was considered positive based on IIFT.

**Table 5:** Cross-reactivity of reactive samples with rS1-ELISA and other human coronaviruses.

	Sample identifier	Whole-virus IIFT IgG titer		rS1-ELISA titer IgG	rS1-IIFT titer IgG				
		229E	SARS	HKU1	229E	OC43	SARS	NL63	HKU1
Reactive BD	BD 2014/597	*≥320*	0	*≥100*	*320*	*320*	0	*3200*	*3200*
	BD 2015/1303	*320*	0	*≥101*	*3200*	*3200*	0	*3200*	*320*
	BD 2015/3004	*≥1000*	0	*≥101*	*320*	*3200*	0	*3200*	*3200*
	BD 2015/3119	*≥320*	0	*≥101*	*320*	*320*	0	*320*	*320*
	BD 2015/3380	*320*	0	*≥101*	*3200*	*3200*	0	*320*	*320*
	BD 2015/3513	*≥1000*	0	*≥101*	*3200*	*3200*	0	*3200*	*3200*
	BD 2015/4435	*≥320*	0	*≥101*	*320*	*3200*	0	*3200*	*3200*
Borderline BD	BD 2012/2644	*≥1000*	0	*≥101*	*320*	*320*	0	*320*	*320*
	BD 2015/1816	*≥320*	0	*≥101*	*3200*	*3200*	0	*3200*	*3200*
	BD 2015/4708	ND	ND	*≥101*	*3200*	*3200*	0	*320*	*3200*
Selected negative BD (showing 3/13)	BD 2015/2859	ND	ND	ND	*3200*	*3200*	0	*3200*	*3200*
	BD 2015/2988	ND	ND	ND	*320*	*320*	0	0	*320*
	BD 2015/3379	*320 (IgM = 0)*	0 (IgM = 0)	*≥101*	*3200*	*3200*	0	*320*	*320*
Reactive CP	CP Mar.2015	*100*	0	*≥101*	*3200*	*320*	*320*	*320*	*320*
	CP May.2015	*1000*	0	*≥101*	*3200*	*3200*	0	*320*	*3200*
	CP Feb.2016	0	*1000*	*≥101*	*3200*	*>10000*	*3200*	0	0

Positive samples are shown in *italic*. ND: not determined.

## Data Availability

The data used to support the findings of this study are available from the corresponding author upon request.
